# Racial and Ethnic Disparities in Diabetes Prevention Outcomes: Insights from the Prediabetes Informed Decisions and Education Study

**DOI:** 10.1089/heq.2023.0186

**Published:** 2024-08-26

**Authors:** Yelba Castellon-Lopez, O. Kenrik Duru, Norman Turk, Gerardo Moreno, Keith C. Norris, Amanda Vu, Rintu P. Saju, Chi-Hong Tseng, Kia Skrine-Jeffers, Carol M. Mangione, Dominick Frosch, Tannaz Moin

**Affiliations:** ^1^ Department of Biomedical Sciences, Cedars-Sinai Medical Center, Cancer Research Center for Health Equity, Samuel Oschin Comprehensive Cancer Institute, Los Angeles, California, USA.; ^2^ David Geffen School of Medicine, University of California, Los Angeles, California, USA.; ^3^ Department of Medicine, Division of General Internal Medicine-Health Services Research, University of California, Los Angeles, California, USA.; ^4^ UCLA School of Nursing, Los Angeles, California, USA.; ^5^ Jonathan and Karin Fielding School of Public Health, University of California, Los Angeles, California, USA.; ^6^ Patient Centered Outcomes Research Institute, Washington, DC, USA.; ^7^ HSR&D Center for the Study of Healthcare Innovation, Implementation, and Policy, Veterans Affairs Greater Los Angeles Health System, Los Angeles, California, USA.

**Keywords:** shared decision making, diabetes prevention, racial and ethnic disparities, evidence-based interventions, weight loss

## Abstract

**Background:**

To achieve health equity, interventions should yield similar effectiveness across all patient subgroups. However, the adoption of diabetes prevention strategies and successful weight loss in “real-world” Diabetes Prevention Program (DPP) translational studies have varied by race and ethnicity. We examined racial and ethnic differences in diabetes prevention outcomes among study participants from the Prediabetes Informed Decisions and Education (PRIDE) Study.

**Methods:**

In a retrospective analysis of data from the PRIDE cluster randomized trial across a large health system, we examined (1) percent weight change and (2) uptake of DPP and/or metformin among overweight/obese participants with prediabetes 12 months after participating in a pharmacist-led shared decision-making (SDM) intervention. We stratified the outcomes by race and ethnicity using a generalized linear mixed-effects model.

**Results:**

The study participants (*n* = 515) had an average age of 56 years (standard deviation [SD] = 11.0), hemoglobin A1c of 6.0% (SD = 0.20), and body mass index of 30.3 (SD = 5.2). Black/African American and Latino study participants lost significantly less weight at the 12-month follow-up compared with White/Caucasian participants (−1.0% and −1.2%, respectively, vs. −3.3%, *p* < 0.01 for both comparisons). There was no significant difference in the adoption of diabetes prevention strategies between racial and ethnic groups after completing an educational SDM intervention.

**Conclusion:**

To better promote health equity, future studies should investigate the potential causal factors for these differences in weight loss, such as variations in socioeconomic status, physical activity, cultural influences, and neighborhood characteristics.

## Introduction

The Diabetes Prevention Program (DPP) trial and other studies have shown that weight loss using structured, year-long, intensive lifestyle behavior change interventions or metformin can significantly lower the risk of incident type 2 diabetes (T2D) among prediabetic patients with high risk of developing diabetes.^1–11^ The landmark DPP trail showed that participants achieved a 58% reduction in the risk of developing T2D regardless of racial and ethnic identity and baseline weight.^2^ Almost half of the participants in the landmark randomized controlled DPP trial were identified as being from racial and ethnic minoritized groups at higher risk for diabetes, and outcomes did not vary by ethnicity or race. However, several “real-world” translational studies of the Centers for Disease Control and Prevention (CDC) that supported National DPP have demonstrated disparities in weight loss for Hispanic and non-Hispanic Black (NHB) participants compared with other racial and ethnic groups.^12–14^

Ensuring equitable outcomes for evidence-based interventions, such as the National DPP, is critical. There have been incredible gains in national policies regarding the coverage of the National DPP. However, younger patient groups and those from racial and ethnic minorities may be less likely to participate, and their outcomes may fall short even when they do.^11–14^ Increasing urgency for action are recent data demonstrating the prevalence and incidence of prediabetes rising among NHB and Hispanic individuals in the United States.^15,16^ Although not all patients with prediabetes develop T2D, the prevalence of diabetes is projected to increase almost threefold by 2060, and both NHB and Hispanic individuals are at a higher risk of progression and some diabetes-related complications.^17–19^ To ensure equitable outcomes in diabetes prevention, we need to ensure that behavioral and lifestyle interventions are similarly effective across all participants. As weight loss is a central predictor of diabetes prevention, it is important to examine real-world racial and ethnic differences in weight change outcomes to develop more targeted interventions and areas of focus. Exploring racial and ethnic differences in the adoption of evidence-based diabetes prevention strategies is important.

Our goal was to examine whether real-world diabetes prevention-focused outcomes among participants in the Prediabetes Informed Decisions and Education (PRIDE) study varied by race or ethnicity. The PRIDE study was a cluster randomized clinical trial (RCT) of shared decision-making (SDM) for diabetes prevention implemented within a large regional network of primary care offices in an academic health system. Investigators of the PRIDE study found that an SDM intervention increased the uptake of DPP lifestyle changes and/or metformin and led to greater weight loss at 12 months among participants in clinics randomized to the SDM intervention compared with matched participants from clinics randomized to usual care.^20^ SDM engages patients in a discussion about the benefits and risks associated with various prevention or treatment options, taking their values and preferences into consideration to help them finalize a clinical decision. Therefore, we hypothesized that there would be no difference in weight change or adoption of evidence-based diabetes prevention strategies based on race or ethnicity after completion of the SDM intervention for diabetes prevention.

## Methods

### Study Design

The PRIDE study was conducted across 20 primary care clinics between 2015 and 2018 within a large regional academic health system that primarily provides care to patients with employer-based insurance or Medicare. It was the first cluster RCT to use SDM to engage patients with prediabetes in evidence-based diabetes prevention strategies. Primary outcomes for the PRIDE study are published in a separate article.^20^ We conducted a retrospective cohort study using electronic medical record (EMR) data from participants receiving care in 10 clinics randomized to deliver SDM (usual care participants from 10 control clinics were not included in this analysis).^20^ Our primary goal was to examine differences in outcomes by race and ethnicity at a 12-month follow-up after SDM completion. The Institutional Review Board of the University of California, Los Angeles, approved this study (IRB#15–000310).

### Study Participants

PRIDE participants met the following inclusion criteria: age 18–74 years, body mass index (BMI) ≥25 kg/m^2^ or ≥23 kg/m^2^ for Asian patients,^21^ and prediabetes (hemoglobin A1C [HbA1c] of 5.7–6.4% within the prior 3 months). Patients with diabetes (i.e., HbA1c > 6.4%, International Classification of Diseases [ICD]-9 or ICD-10 billing codes for diabetes, and/or use of oral antiglycemic medications or insulin), polycystic ovarian syndrome, advanced chronic kidney disease (estimated glomerular filtration rate ≤45 mL/min/1.73 m^2^), active eating disorder(s), dementia, active cancer diagnosis, congestive heart failure, or pregnancy within the year were excluded. The PRIDE recruitment team used EMR data to identify 2,344 potentially eligible participants, of which 583 were excluded and 1,761 were confirmed eligible and were mailed study invitation letters. Of those participants confirmed eligible, 515 opted for SDM visits (29% of those who received letters overall and 48% of those reached by phone).^20,22,23^ To examine differences in outcomes stratified by race and ethnicity, we used the entire sample of patients who completed the SDM intervention.

### SDM Intervention

Participants met one-on-one in a private room with a clinical pharmacist during SDM intervention. All pharmacists were trained clinical pharmacists embedded in the primary care clinic setting with experience in managing diabetes and other chronic diseases. All pharmacists received training in motivational interviewing and SDM delivery prior to the study to ensure fidelity and promote standardization of the SDM intervention. The dyad jointly reviews an online decision aid called “Prediabetes: Which Treatment Should I Use to Prevent Type 2 Diabetes?” The decision aid was created by Healthwise^®^, a well-established provider of patient support tools and health information for over 35 years.^24^ The decision aid compared the two evidence-based options for diabetes prevention (lifestyle changes through the DPP and metformin use). Using data from the existing scientific literature, the decision aid summarized the average risk of progression to T2D in 3 or 10 years with lifestyle changes, metformin, or no action.

During the visit, the participants were asked if they wanted to participate in the DPP lifestyle intervention and initiate metformin (or both strategies). Participants also elected to take no action. Pharmacists prescribed metformin after obtaining PCP approval for those who elected metformin. Those who elected the DPP lifestyle intervention were referred to a local CDC-recognized DPP. Participant outcomes, including weight loss and metformin uptake and/or the DPP lifestyle intervention, were tracked for 12 months after the SDM session. We included all SDM participants irrespective of their selected choice (DPP, metformin, both, or neither).

### Variables

We measured the outcomes of interest among all participants with prediabetes who completed the SDM intervention (*n* = 515): (1) percent weight loss at the 12-month follow-up, measured as a continuous variable, and (2) uptake of diabetes prevention strategies, including uptake of DPP, metformin, or both after SDM, measured as a dichotomous variable (Y/N). Based on CDC guidelines, we defined DPP uptake as attendance in ≥9 sessions in the 12-month CDC-recognized DPP, as confirmed by the DPP provider.^25^ We assessed metformin uptake based on patient-reported metformin use during medication reconciliation at any clinical visit using EMR documentation. Categorical variables were used for self-reported race and ethnicity: non-Hispanic Asian American/Pacific Islander (AAPI), non-Hispanic Black (Black/African American), Hispanic (Latino), non-Hispanic White (White/Caucasian), and Other (including multiracial, Alaskan Native, and non-Hispanic Native American). Other covariates included patient-level demographics and clinical measures, including self-reported annual income and data from the EMR, such as BMI, HbA1c, estimated glomerular filtration rate, insurance coverage (Medicare, private insurance, Medi-Cal, or self-pay), frequency of baseline visits, use of any prescription medication for weight loss, and number of comorbidities. Additional covariates measured in the EMR included dichotomous variables for anxiety, atrial fibrillation, cerebrovascular accident, chronic obstructive pulmonary disease, coronary artery disease, depression, hypertension, hyperlipidemia, peripheral artery disease, osteoarthritis, and substance use disorders.

### Analysis

We calculated the descriptive statistics of the study variables, including means, standard deviations, and univariate frequencies. We used *t*-tests and chi-squared tests to evaluate continuous and categorical bivariate associations between the study variables and primary predictors, race, and ethnicity. We used two generalized linear mixed-effects models to compare the (1) percent weight change at the 12-month follow-up by race and ethnicity and (2) successful uptake of a diabetes prevention strategy (metformin and/or DPP) 12 months after SDM by race and ethnicity. Adjusted estimates of the percent change from baseline weight were generated via repeated-measures mixed models that accounted for clinic clustering, baseline weight, income, age, gender, race and ethnicity, and the number of DPP sessions attended. We also conducted a sensitivity analysis by adding an interaction term for race and ethnicity based on DPP attendance to the weight loss models to account for possible differential attendance among ethnic and racial groups as a predictor of weight loss.^12,26^

We used multiple imputations to impute missing race, ethnicity (4.0% missing), income (16.0% missing), and 12-month weight (12.0% missing) data. The imputation model included the variables listed in Table 1. We also included 4-month weight measurements in the imputation models for missing 12-month weights. A significance level of α = 0.05 was used for all analyses, which were conducted with SAS, version 9.4 (SAS Institute).

**Table 1.** Baseline Characteristics by PRIDE Study Intervention Participants (*N* = 515)

Baseline characteristics	White *N* = 202	Black *N* = 74	Latino *N* = 86	AAPI *N* = 95	Other *N* = 36	Missing *N* = 22	*p*-Value^a^
Mean Age (SD)	58.7 (10.9)	57.4 (11.2)	52.4 (11.3)	50.4 (10.9)	56.6 (10.9)	53.8 (9.7)	<0.01
Female	52.5%	64.9%	62.8%	52.6%	58.3%	36.4%	0.11
Income^b^							
	14.2%	27.7%	19.7%	5.1%	12.5%	16.7%	<0.01
$45,000–84,999	28.4%	26.2%	30.3%	20.2%	34.4%	11.1%	
$85,000+	57.4%	46.2%	50.0%	74.7%	53.1%	72.2%	
Mean BMI, kg/m^2^ (SD)	30.4 (4.8)	31.6 (5.5)	31.5 (5.3)	27.5 (4.3)	31.6 (5.9)	30.3 (4.4)	<0.01
HbA1c, % (SD)	5.9 (0.20)	6.0 (0.21)	6.0 (0.19)	6.0 (0.17)	6.0 (0.22)	5.9 (0.17)	0.04
eGFR							
>89	14.8%	28.4%	37.2%	39.0%	16.7%	36.4%	<0.01
60–89	70.3%	68.9%	58.1%	60.0%	72.2%	63.6%	
45–69	14.8%	2.7%	4.6%	1.0%	11.1%	0.0%	
Medicaid (Y/N)	2.5%	12.2%	4.6%	0.0%	2.8%	0.0%	<.001
Medicare (Y/N)	30.2%	33.8%	10.5%	9.5%	25.0%	13.6%	<0.01
Number of Comorbidities							
0	16.8%	21.6%	38.4%	32.6%	27.8%	50.0%	<0.01
1	29.2%	33.8%	19.8%	36.8%	25.0%	13.4%	
2	27.2%	12.2%	24.4%	21.0%	38.9%	18.2%	
3+	26.7%	32.4%	17.4%	9.5%	8.3%	18.2%	
Hypertension	37.6%	46.0%	27.9%	30.5%	52.8%	13.6%	<0.01
Hyperlipidemia	55.0%	47.3%	40.7%	43.2%	50.0%	27.3%	0.06
CAD	4.0%	0.0%	3.5%	1.0%	0.0%	0.0%	0.24
AFib/Arrhythmia	7.4%	4.0%	4.6%	6.3%	0.0%	9.1%	0.51
COPD	16.8%	16.2%	11.6%	13.7%	11.1%	18.2%	0.83
CVA	3.5%	1.4%	5.8%	2.1%	0.0%	0.0%	0.37
PVD	1.5%	0.0%	0.0%	0.0%	0.0%	0.0%	0.46
Osteoarthritis	12.4%	18.9%	7.0%	4.2%	13.9%	9.1%	0.04
Depression	20.3%	16.2%	10.5%	4.2%	0.0%	18.2%	<0.01
Anxiety	16.8%	10.8%	11.6%	2.1%	2.8%	4.6%	<0.01
Substance Abuse	4.5%	5.4%	4.6%	1.0%	0.0%	9.1%	0.32
Frequency baseline visits							
0–1	8.4%	14.9%	11.6%	24.2%	25.0%	45.4%	<0.01
2	12.9%	14.9%	14.0%	13.7%	8.3%	9.1%	
3	11.4%	12.2%	11.6%	19.0%	19.4%	9.1%	
4+	67.3%	58.1%	62.8%	43.2%	47.2%	36.4%	
Use of Weight Loss Rx	5.4%	5.4%	2.3%	1.0%	2.8%	4.6%	0.48

^a^ Chi-squared tests of significance were used to compare categorical variables, and ANOVA was used to compare continuous variables.

^b^ *N* = 79 participants had missing income.

AAPI, non-Hispanic Asian/Pacific Islander; AFib, atrial fibrillation; Black, African American/non-Hispanic Black; BMI, body mass index; CAD, coronary artery disease; COPD, chronic obstructive pulmonary disease; CVA, cerebrovascular accident; eGFR, estimated glomerular filtration rate; HbA1c, hemoglobin A1C; PRIDE, Prediabetes Informed Decisions and Education; PVD, peripheral vascular disease; Rx, prescription; SD, standard deviation; White, non-Hispanic White.

## Results

Participants (*n* = 515) were 56 years old on average (SD = 11), with a mean HbA1c of 6.0% (SD = 0.20) and a BMI of 30.3 kg/m^2^ (SD = 5.2; Table 1). More than half of the participants reported an income of ≥$85,000 (57.9%) and were female (55.7%). Most participants self-identified as White/Caucasian (39.2%), followed by AAPI (18.4%), Latino (16.7%), Black/African American (14.4%), and Other (7.0%), and 4.3% did not report their race and/or ethnicity. There were significant baseline differences in BMI, age, and income across ethnic and racial groups (Table 1).

### Percent Weight Loss

At the 12-month follow-up, the mean percentage weight change across all PRIDE participants was −2.5% (SD = 5.39), but this differed by race and ethnicity in our adjusted models (Table 2). Latino (−1.1% change, *p* = 0.007) and Black/African American (−1.0% change, *p* = 0.004) participants lost significantly less weight compared with White/Caucasian participants, who lost 3.3% body weight on average. There was no significant difference in the percentage of weight loss between AAPI and White/Caucasian participants. There was no statistical difference in missing weight between White/Caucasian, Black/African American, AAPI, and Latino participants (*p* = 0.068). Increased DPP session attendance was a significant predictor of weight loss (*p* < 0.001). Self-reported income and baseline weight were not predictors of percent weight loss in this study. Among people who completed at least one session, there was no difference in attendance by race and ethnicity. A sensitivity analysis, with an interaction between ethnicity, race, and DPP session attendance, did not change the magnitude or significance of the main outcome.

**Table 2.** Adjusted Weight Change over 12 Months^a^

Race/ethnicity	Baseline weight (lbs; 95% confidence interval)	% Weight change (95% confidence interval)	*p*-Value^b^
AAPI (*N* = 95)	166.1 (157.8, 174.3)	−2.5 (−3.7, −1.2)	0.28
Black (*N* = 74)	207.2 (199.4, 215.0)	−1.0 (−2.4, 0.4)	0.004
Latino (*N* = 86)	192.3 (185.0, 199.6)	−1.2 (−2.4, 0.1)	0.007
White (*N* = 202)	197.8 (192.6, 203.0)	−3.3 (−4.1, −2.4)	—
Other (*N* = 36)	198.3 (187.2, 209)	−1.9 (−3.6, −0.1)	0.17

Missing race/ethnicity, income, and 12-month weight changes were also imputed.

^a^ Reported weight is controlled for age, baseline weight, clinic, income, number of DPP sessions attended, and sex.

^b^ We used White as the reference category for % weight loss pairwise *t*-test comparisons.

DPP, Diabetes Prevention Program; lbs, pounds.

### Successful Diabetes Prevention Strategy Uptake

Among the 515 study participants who completed the SDM intervention, 32.8% had adopted metformin and/or DPP lifestyle changes. Our adjusted models found no significant difference in the use of a diabetes prevention strategy (metformin and/or DPP lifestyle changes) by ethnicity or race (*p* = 0.32, Table 3).

**Table 3.** Adjusted Uptake of Diabetes Prevention Strategy by Race/Ethnicity^a^

Race/ethnicity^b^	% Uptake of prevention strategy	*p*-Value^c^
NHA/PI (*N* = 95)	29.0 (17.7, 40.4)	0.32
NHB (*N* = 74)	30.2 (18.9, 41.6)	
Hispanic (*N* = 86)	39.4 (27.8, 51.1)	
Other (*N* = 36)	24.2 (9.7, 38.7)	
NHW (*N* = 202)	33.4 (25.5, 41.4)	—

^a^ Diabetes prevention strategies include lifestyle changes and the use of metformin. The analysis was controlled for age, clinic type, income, and sex. Race/ethnicity and income were imputed or missing.

^b^ NHW is the reference category for % uptake pairwise *t*-test comparisons.

^c^ There were no significant differences in percent uptake between NHW and any other race/ethnic category (α = 0.05) when examining pairwise *t*-tests.

NHA/PI, non-Hispanic Asian/Pacific Islander; NHB, non-Hispanic Black; NHW, non-Hispanic White.

## Discussion

Our study found that the percentage of weight loss at 12 months was lower among Latino and Black/African American participants than among White/Caucasian participants, but there was no difference in the uptake of diabetes prevention strategies by race and ethnicity after SDM. Our results are the first to demonstrate racial and ethnic disparities in weight loss persisted even after controlling for the number of DPP sessions attended. We did not find any difference in the rates of uptake of diabetes prevention strategies (i.e., metformin and/or DPP lifestyle change) by ethnicity/race, which suggests that among our insured, middle-class sample, barriers to initial DPP enrollment or metformin use (i.e., uptake) may be lower than those associated with long-term adoption (i.e., implementation of lifestyle changes after DPP enrollment). Our findings highlight the importance of evaluating the specific causes of racial and ethnic differences in weight outcomes among high-risk prediabetes groups. Understanding the factors contributing to these differences merits additional investigation to work toward health equity in T2D prevalence and outcomes.

In light of the evidence that real-world adaptations of diabetes prevention efforts do not provide equal benefits to all racial and ethnic subgroups, it is essential to identify specific strategies to improve outcomes in Black/African American and Latino patients. Although this study did not demonstrate the elimination of weight loss disparities after adjusting for DPP attendance, finding effective strategies to maintain attendance and retention throughout the 12-month program remains a key goal because other studies have found a dose-dependent weight-loss response.^2,9^ In a large cross-site DPP evaluation, Clennin et al. identified that weight loss disparity among Latino and Black/African American participants was due to lower program participation and lower levels of self-reported physical activity, respectively.^13^ Another recent comprehensive evaluation of National DPP data indicated that attending more DPP sessions was associated with greater weight loss for all racial and ethnic groups, although Latino and Black/African American individuals were less likely to achieve the 5% weight loss goal than their White/Caucasian counterparts.^12^ Several other studies have confirmed the link between DPP attendance and weight loss,^2,12,26,27^ and our team has previously shown that DPP attendance among individuals who received SDM for diabetes prevention was associated with higher rates of patient activation.^28^ However, our findings showed a significant weight loss disparity for Latino and Black/African American participants even after controlling for attendance. This suggests that there may be other factors, outside of individual level factors that should be explored.

Identifying the underlying causes of weight loss disparities for racial and ethnic minoritized groups is necessary for the development of targeted interventions that promote health equity. Structural factors can have a significant role in shaping the prevalence of obesity and therefore prediabetes risk within communities^29^ and for some racial and ethnic groups.^30,31^ These factors encompass a wide range of environmental, economic, and social elements that influence individuals’ lifestyle choices and behaviors related to diet and physical activity. For instance, the availability and accessibility of healthy foods versus high-calorie processed options in neighborhoods can greatly impact dietary habits. Additionally, built environments, such as the presence of sidewalks, parks, and recreational facilities, influence opportunities for physical activity.^32^ Institutional racism is also linked to differential health outcomes for racial and ethnic minorities.^33^ A prior qualitative study of PRIDE participants revealed that Latino and Black/African American participants more often endorsed weight loss barriers of limited time to make lifestyle changes due to long work and commuting hours, inconvenient DPP class locations and offerings, and limited disposable income for extra weight loss activities (i.e., gym memberships and exercise equipment).^34^ Understanding and addressing these structural factors are essential in developing effective public health interventions to combat obesity and promote diabetes prevention for all groups.

Achieving health equity in diabetes prevention interventions for populations that have been historically marginalized will require a shift in translational diabetes research. Such research should focus on interventions beyond individual factors, such as knowledge, attitudes, and behaviors, to address the root causes of existing disparities in diabetes outcomes. Future interventions should focus on addressing the impact of the social determinants of health. Existing literature has demonstrated that socioeconomic status, living and working conditions, food environment quality, sociocultural and sociopolitical contexts, and life course exposures contribute to population-specific diabetes outcomes and health disparities. Future diabetes prevention efforts should target the social determinants of health and aim to change those factors through multisector partnerships that promote coordinated action across local and national sectors to eliminate population-level disparities.

Our study has some limitations. We conducted the study within one large health system, which limits the generalizability of our findings, although the health system is situated in one of the most diverse and populous countries in the nation. In addition, income was self-reported and, therefore, may have been understated or overstated. Finally, we used multiple imputations to impute missing 12-month follow-up weights; however, this was done for only a small portion of our study participants (12.0%).

Equitable diabetes prevention efforts are a public health imperative, given the decreased quality of life, burden of complications, and economic costs associated with incident diabetes in ethnically diverse populations such as Black/African Americans and Latinos.^35–38^ The rising burden of diabetes is greatest in racial and ethnic minoritized groups that already suffer from inequities in health and health care. Despite similar rates of adoption of diabetes prevention strategies after SDM, we found that Latino and Black/African American participants lost less weight at the 12-month follow-up than White/Caucasian participants, even after controlling for income and DPP lifestyle change attendance. National diabetes prevention initiatives should add racial equity as a key objective to encourage care interventions and research to better understand and address racial and ethnic gaps in the desired outcomes. Future studies should also explore the potential causes for these disparities, potentially incorporating structural environmental factors, social determinants, and/or cultural barriers.

## Data Access Statement

Datasets used and/or analyzed during this study are available from the corresponding author upon reasonable request.

## Abbreviations Used

AAPI: Asian American/Pacific Islander
BMI: body mass index
CDC: Centers for Disease Control and Prevention
DPP: Diabetes Prevention Program
EMR: electronic medical record
HbA1c: hemoglobin A1c
NHB: non-Hispanic Black
PRIDE: PRediabetes Informed Decisions and Education
RCT: Randomized clinical trial
SDM: shared decision-making (SDM)
T2D: type 2 diabetes
